# Dietary Profiles, Nutritional Biochemistry Status, and Attention-Deficit/Hyperactivity Disorder: Path Analysis for a Case-Control Study

**DOI:** 10.3390/jcm8050709

**Published:** 2019-05-18

**Authors:** Liang-Jen Wang, Ya-Hui Yu, Ming-Ling Fu, Wen-Ting Yeh, Jung-Lung Hsu, Yao-Hsu Yang, Hui-Ting Yang, Shih-Yi Huang, Ien-Lan Wei, Wei J. Chen, Bor-Luen Chiang, Wen-Harn Pan

**Affiliations:** 1 Department of Child and Adolescent Psychiatry, Kaohsiung Chang Gung Memorial Hospital and Chang Gung University College of Medicine, Kaohsiung 83301, Taiwan; wangliangjen@gmail.com; 2 Institute of Epidemiology and Preventive Medicine, College of Public Health, National Taiwan University, Taipei 10617, Taiwan; yahui.doris.yu@gmail.com (Y.-H.Y.); wjchen@ntu.edu.tw (W.J.C.); 3 Department of Food and Nutrition, Chung-Hwa University of Medical Technology, Tainan 71703, Taiwan; 207linto@pchome.com.tw; 4 Institute of Biomedical Sciences, Academia Sinica, Taipei 11529, Taiwan; wenting@ibms.sinica.edu.tw; 5 Department of Neurology, Chang Gung Memorial Hospital Linkou Medical Center and College of Medicine, Neuroscience Research Center, Chang-Gung University, Linkou, Taoyuan 33305, Taiwan; tulu@ms36.hinet.net; 6 Graduate Institute of Mind, Brain, and Consciousness, Taipei Medical University, Taipei and Brain and Consciousness Research Center, TMU Shuang Ho Hospital, New Taipei City 11031, Taiwan; 7 Department of Pediatrics, National Taiwan University Hospital, College of Medicine, National Taiwan University, Taipei 10617, Taiwan; yan0126@ms15.hinet.net (Y.-H.Y.); gicmbor@ntu.edu.tw (B.-L.C.); 8 Department of Food Safety, Taipei Medical University, Taichung City 40402, Taiwan; lulu0319@mail.cmu.edu.tw; 9 School of Nutrition and Health Sciences, Taipei Medical University, Taipei 11031, Taiwan; sihuang@tmu.edu.tw; 10 School of Nursing, National Yang-Ming University, Taipei 11221, Taiwan; weinur@ym.edu.tw; 11 Department of Biomedical Science and Technology, College of Life Science, National Taiwan University, Taipei 10617, Taiwan

**Keywords:** ADHD, diet, nutritional biochemistry, fatty acid profile, vitamin, mineral

## Abstract

This study aims to investigate dietary and nutritional biochemistry profiles of attention-deficit/hyperactivity disorder (ADHD) and to explore their potential relationship by path analysis. We enrolled 216 children with ADHD and 216 age-, height- and gender-matched controls from 31 elementary schools in Taiwan. Dietary intake of the participants was assessed using a food frequency questionnaire (FFQ). Fasting blood samples were collected to determine the serum levels of multiple nutritional markers. Moreover, we employed a structural equation model (SEM) to link diet, nutritional markers and ADHD. Compared to healthy control, ADHD children had significantly lower serum levels of vitamin B12, folate, vitamin B6, ferritin concentration, and monounsaturated fatty acids (MUFA), but higher levels of serum saturated fatty acids (SFA), n-6/n-3 fatty acid ratio, and inorganic phosphorous concentration. Children with ADHD had more intake of nutrient-poor foods such as high sugar and high fat foods, and had less intake of vegetable, fruit, protein-rich foods than their counterpart. SEM analysis showed that the poor nutritional biochemistry profiles linked the association between unhealthy dietary patterns and ADHD. In conclusion, an unhealthy dietary pattern may be a predecessor of the poor nutritional biochemistry status, and managing diet and nutrition conditions should be considered to improve ADHD symptoms in children.

## 1. Introduction

Attention-deficit/hyperactivity disorder (ADHD), a psychiatric disorder commonly found in children and adolescents, is characterized by inattention, hyperactivity, and impulsivity [1]. This disorder affects 3% to 10% of school-age children worldwide [2] and a local prevalence rate of 7.5% was reported in a study of Taiwan [3]. Although ADHD is generally considered as a highly genetic disorder, attention has been raised to the potential role of ‘unhealthy’ diets or nutrient deficiency in the susceptibility of ADHD [4,5,6]. Some earlier studies showed that some food additives (colorings, flavorings and preservatives) may increase hyperactivity in children with behavior problems [4] and elimination diet (exclusion of food additives, gluten or casein) has been suggested as a treatment for ADHD and autism spectrum disorder [7]. Recently, evidences have indicated potential roles of nutrient poor diets on learning and behavior in children [5,8].

Data from Nutrition and Health Survey in Taiwan 2001–2002 showed that children with a dietary pattern of more free sugar-rich foods and/or fried foods, but less nutrient-dense foods (vegetable, fruit, fish, egg, meat, and dairies) had poorer overall school performance compared to their counterparts [9]. Similarly, in community-dwelled Korean children; high intake level of sweets, fried foods, and salt has been positively associated with difficulty in learning, attention deficits, and behavioral problems [10]. Specifically, increased risk of ADHD has been associated with a western-style diet, high in fat and refined sugars. On the contrary, a “healthy diet” such as the Mediterranean diet (abundant in fruits, vegetables, whole grains, legumes, seafood and olive oil) [11] or some other dietary patterns rich in fiber, folate, and ω-3 fatty acids have been inversely associated with ADHD [6]. In addition, it has been shown that the association between ADHD and poor dietary pattern was present irrespective of medication use [12].

A critical question is whether poor nutritional status plays a role in ADHD etiology. A study indicated that nearly half of the ADHD children had suboptimal nutrition, while only 11.1% of typically developed non-ADHD children revealed such a condition [13]. Studies have suggested a role of vitamin B12 and vitamin D in the pathogenesis of mental disorders in childhood and adolescence [14,15]. Ferritin deficiency has been associated with ADHD in children [16,17]. A study in Taiwan suggested that blood profiles with respect to iron, vitamin C, and fatty acids were different between children with ADHD and those without [18]. However, nutrient-based dietary intervention studies are limited [19]. Among them, supplementation of poly-unsaturated ω-3 fatty acid were most studied. Although it has yielded a consistent effect, the effect is relatively small. Therefore, ω-3 fatty acid alone is not considered as a promising approach for ADHD treatment [20].

We hypothesized that the pathophysiology of ADHD may be related to nutritional imbalance and involved in multiple dietary components which is in part a result of an unhealthy dietary pattern. Carrying out a case-control study and applying a path analysis, we aim to not only document the dietary and nutritional biochemistry difference between the ADHD and the control children, but also explore the potential inter-relationships among diet, nutritional biochemistry, and ADHD.

## 2. Materials and Methods

### 2.1. Study Subjects

This study first received approval from the Medical Research Ethical Committee, Institute of Biomedical Sciences, Academia Sinica (AS-IBMS-MREC-92-01). The subject’s recruitment and study procedures have been described elsewhere [21]. We mailed invitation letters to elementary schools located in the larger Taipei metropolitan area and visited the schools that agreed to participate. Once the teachers distributed the informed consent forms and obtained signed forms from the parents, we screened the participating classes for ADHD students using two validated scales: the Chinese version of the Conners Teacher Rating Scale (CTRS) (for teachers) and the Chinese version of the Werry-Weiss-Peters Activity Scale (WWPAS) (for parents) [22]. Of all the students, 287 students scored higher than the 85th percentile values on both scales and were referred for further clinical evaluation. After being interviewed by a child psychiatrist of our research team, 252 students were diagnosed with ADHD according to the criteria of the Diagnostic and Statistical Manual, Fourth Edition (DSM-IV). The control students were then randomly selected from the same class of the ADHD students among those of the same sex who also had the closest age, height, and body weight (within a 5% range). Once students with incomplete data were excluded, this analysis included a total of 216 children with ADHD and 216 controls.

### 2.2. Measurements

#### 2.2.1. Demographics and Dietary Assessment

We obtained information about the education levels, family income, lifestyle, and disease history of the students and their parents through a questionnaire interview. Meanwhile, the body weights and heights of the participating children were measured by trained technicians. We asked parents to fill out a food frequency questionnaire (FFQ) to assess the dietary intake of the child in the previous year [23].

#### 2.2.2. Food Intake Frequency Assessments

We used food frequency questionnaires [9] filled by parents to assess food consumption frequency (times/per week) of children. The questionnaire contained 12 high-quality foods categories including vegetables, fruits, milk, yogurt, meat, poultry, pork, beef, fish, soy milk, soy products, eggs; while eight low-quality foods categories including fried food, ice cream, sugary and high-fat foods, high-fat snacks, instant noodles, sugar drinks, shaved ice desserts, candy and chocolate.

#### 2.2.3. Food Preference Assessments

The food preference of children was collected by directly interviewing children. The food items of the questionnaire were composed of 20 items which were the same as the items in the food frequency questionnaire. There were three levels of preference: disliking, neutral on, and liking the food (Supplementary Table S1).

#### 2.2.4. Nutritional Biomarker Assessment

Approximately 10 mL venous blood was drawn from each student, which was carefully kept in darkness as much as possible in the process of centrifugation and aliquoting. All the serum and plasma samples were stored at −70 °C until analysis. The plasma pyridoxal 5′-phosphate (PLP) concentration was measured using high performance liquid chromatography (HPLC) [24]. Plasma vitamin B12, folate and ferritin levels were assessed by Chemiluminescent immunoassay (IMMULITE 2000 analyzer, Diagnostic Products Corporation, Los Angeles, California, USA) [25,26,27]. Serum sodium, potassium, calcium, inorganic phosphorus and magnesium concentrations were analyzed by Olympus AU 600 Autoanalyzer (Olympus Corp., Tokyo, Japan). Serum crude fat was extracted by Blight and Dyer method [28] and fatty acids profiles were analyzed by gas chromatography (Thermo electron corporation, Waltham, Massachusetts, USA) [29].

### 2.3. Statistical Analysis

The G-Power 3 software was used for estimating sample size in this matched case-control study. Given the significance level (α) was set at 0.05 and odds ratio (OR) was set as 2.5, 131 cases and 131 controls were required to achieve a power (1-β) of 0.8, and 211 cases and 211 controls were required to achieve a power of 0.95.

All statistical analyses were carried out using the statistical software package SPSS, version 24.0 (SPSS Inc., Chicago, IL, USA) and (IBM SPSS Amos 24.0). Variables being offered as either mean (±standard deviation) or frequency (%), and *p* < 0.05 were considered statistically significant. We performed group comparisons between cases and controls using Chi-square tests for categorical variables and *t*-tests for continuous variables.

To sort out interrelations among large numbers of dietary and nutritional markers, we used principal component analysis (PCA) to identify latent components from either nutritional biochemistry markers or from food frequency variables. Varimax rotation was applied and orthogonal components with Eigen value greater than 1.0 were chosen. Loading scores were used to weight the chosen biochemical or food frequency variables within each PCA component to generate factor score and the variables with loading scores greater than 0.50 were designated as the major contributors of the components. Subsequently, the relationships between ADHD, the dietary factors and nutritional biochemistry factors were determined using the General Linear Model to control for parents’ education levels and balanced budget status.

This study applied Pearson’s correlation to analyze the mutual relationships between the nutritional biochemistry factors and food factors yielded by PCA. Moreover, this study used a structural equation modeling (SEM) to find causal relationship and the strength of the relationships between food frequency, nutritional biochemistry markers, and ADHD. SEM, which is better than traditional methods (e.g., multivariate regression) in analyzing complex causal relationships among variables [30]. The maximum likelihood estimation of model parameters was used to conduct SEM.

## 3. Results

The ADHD children and the control subjects were comparable with respect to age, height, weight, body mass index (BMI), and gender distribution (Table 1). Compared to the control children, ADHD children showed significantly lower serum levels of vitamin B12, folate, and vitamin B6. For minerals, ADHD children exhibited lower serum ferritin concentration, but higher serum inorganic phosphorous (Pi) concentration. ADHD children had a significantly lower proportion of monounsaturated fatty acids (MUFA), but higher proportion of saturated fatty acids (SFA) and polyunsaturated (PUFA) as well as higher n-6/n-3 fatty acid ratio.

Food consumption frequency data were also compared between the ADHD children and the controls (Table 1). Significantly lower average frequency of nutrient-dense foods (vegetable, fruit, milk, meat, poultry, fish, soy milk and eggs), but higher frequency of nutrient-poor foods (fried foods, ice cream, high-fat snacks, instant noodles, sugar drinks, shaved ice desserts, candy and chocolate) was observed in ADHD children compared to the control. Food preference result is consistent with the frequency in that ADHD children more so preferred the nutrient-poor foods, but less so the nutrient-dense foods than their control counterpart (data shown in Supplementary Table S1).

Table 2 shows four nutritional biochemistry factors and four dietary factors derived by PCA that yielded eigenvalues exceeding 1.00, in which loading values for all variables are presented. The Biochemistry factors were respectively named according to the variables selected. Factor 1 primarily consisting of MUFA combined inversely with SFA was therefore labelled “MUFA/SFA Fatty Acids”. Factor 2 containing vitamin B6, B12, and folate, all in the same direction, was labelled “B-Vitamins”. The main components of Factor 3 was ferritin combined inversely with inorganic phosphorus and was named “Minerals”. Factor 4 is comprised of the n-6/n-3 FA Ratio and therefore named directly as n-6/n-3 FA Ratio.

For the four PCA-yielded dietary factors, factor 1 primarily consisting of all nutrient-poor foods was labelled “Nutrient-Poor Foods”. Factor 2 contained vegetable and fruit and was named “Vegetable-Fruit”. The main component of Factor 3 was meat, poultry and fish, and was therefore labelled “Flesh foods”. Factor 4 is comprised soy milk and eggs and was named “Soymilk-Egg”. The relationships between ADHD, the dietary factors and nutritional biochemistry factors were shown in Supplementary Table S2. After controlling for potential socio-demographic confounders (parents’ education levels and balanced budget status), the four dietary factors and four nutritional biochemistry factors were still significantly correlated to ADHD.

Correlation coefficients among the four biochemistry factors and the four dietary factors were shown in Table 3. Nutrient-Poor Foods had a negative correlation with “B-Vitamins” (*r* = −0.24, *p* < 0.001) and “Minerals” (*r* = −0.21, *p* < 0.001). Vegetable-Fruit revealed a positive correlation to “B-Vitamins” (*r* = 0.22, *p* < 0.001) and “Minerals” (*r* = 0.20, *p* < 0.001). “Flesh food” intake was also positively correlated with “B-Vitamins” (*r* = 0.10, *p* = 0.039) and “Minerals” (*r* = 0.11, *p* = 0.022). Finally, “Soymilk-Egg” consumption had a positive correlation with “Minerals” (*r* = 0.12, *p* = 0.016), and negative correlation to n-6/n-3 saturated fatty acids ratio (*r* = −0.12, *p* = 0.013). “MUFA/SFA Fatty Acids” did not have significant correlations to any diet factor.

**Figure 1:**
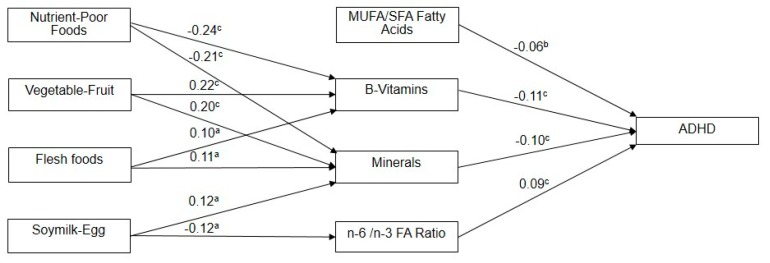
Model for demonstrating the path analysis from diet factors, nutrition factors to a diagnosis of ADHD. MUFA: monounsaturated fatty acids; SFA: saturated fatty acids; ^a^ *p* < 0.05, ^b^ *p* < 0.01, ^c^ *p* < 0.001.

The results of the path analysis of diet, nutritional biochemistry, and ADHD are provided in Figure 1. The aforementioned correlation between dietary and Biochemical factors remained similar and significant in the SEM model. In addition, “MUFA/SFA Fatty Acids” (*β* = −0.06, *p* = 0.005), “B-Vitamins” (*β* = −0.11, *p* < 0.001), “Minerals: ferritin/Pi” (*β* = −0.10, *p* < 0.001) were significantly and inversely associated, but “n-6/n-3 FA Ratio” (*β* = 0.09, *p* < 0.001) was positively associated with having an ADHD diagnosis.

## 4. Discussion

Compared to healthy control children, ADHD children showed significantly lower blood levels of vitamin B12, folate, and vitamin B6. In addition, ADHD children exhibited lower serum ferritin concentration, but higher serum inorganic phosphorous (Pi) concentration. Furthermore, ADHD children had significantly higher SFA proportion and n-6/n-3 fatty acid ratio, but lower MUFA proportion.

With respect to vitamin status, previous studies have suggested a role of vitamin B12 and vitamin D in the pathogenesis of mental disorders in childhood and adolescence [14,15]. In addition, poor B-vitamins status has been associated with not only the ADHD diagnosis but also the symptom severity [31]. Vitamins B2, B6, B12, and folic acid may be part of underlying pathophysiology of ADHD, since they are coenzymes participating in the one-carbon metabolism and contributing to the provision of methyl group which is required for the formation of multiple neurotransmitters including serotonin [32]. Protein-rich foods such as milk, pork, and beef are good sources of vitamin B2, B6, and B12, whereas vegetables and fruits are good sources of folate.

For mineral status, ADHD children exhibited lower serum ferritin concentration, but higher serum Pi concentration. Evidence has shown that ferritin deficiency is associated with ADHD in children [16,17,33]. Although iron deficiency is one major cause of microcytic anemia, contributing to the disruption of the oxygen supply [34]; literature has also shown that iron is involved in neurotransmitter production and iron deficiency may disturb cognitive ability [35]. Aforementioned pathophysiology may explain the ADHD characteristics such as inattention or forgetfulness. To the best of our knowledge, this is the first study to reveal that relation between high serum Pi levels and ADHD. As for serum biochemistry, high levels of phosphorus can negatively affect the ability to effectively use other minerals, such as iron, calcium, magnesium, and zinc [36]. Phosphorus is a mineral widely appearing in all natural foods. However, a high content of inorganic phosphate is seen in sugar-containing beverages and many different types of processed foods such as thickening agent, color stabilizer, and flavor enhancer [37]. The elevated Pi levels may be associated with an excessive intake of food products containing inorganic phosphate. It is not clear whether Pi is a marker of these nutrient-poor foods or itself has direct effects on ADHD etiology.

Furthermore, ADHD children had significantly lower MUFA proportion in blood lipids, but slightly higher SFA and PUFA proportions. The advantages of MUFA over SAF has been widely documented [38]. The ω-6/ω-3 fatty acid ratio was higher in ADHD children in a meta-analysis study [39] which is in line with the results in our study. The findings suggest either adverse effects of ω-6 fatty acids or beneficial effects of ω-3 fatty acids. Previous studies have demonstrated that children with ADHD and autism spectrum disorders had low levels of EPA, DHA, but high levels of AA as well as a high ratio of ω-6/ω-3 PUFA and these profiles correlated significantly with symptoms [40]. Beneficial effects of ω-3 PUFA on various inflammatory diseases have been well documented. Dietary supplementation with ω-3 PUFAs improves both visual acuity and the RBC fatty acid profile in school-age children with lower IQs or ADHD [41]. It has also been proposed to balance between ω-3 and ω-6 fatty acids for overcoming many non-communicable diseases including cardiovascular health [42] and dyslexia [43].

It is important to understand the diet-biochemical profile-ADHD interrelationships. We found ADHD children prefer nutrient-poor foods (fried foods, ice cream, high-fat snacks, instant noodle, sugar drinks, shaved ice desserts, candy and chocolate), but not nutrient-dense foods (vegetable, fruit, milk, meat, poultry, fish, soy milk and eggs). Their dietary pattern is consistent with the food preference. Previous studies have revealed that a higher intake of sweets, fried food, and salt is positively associated with ADHD, but a balanced diet is inversely associated [6,10]. Our findings generally support this point of view. However, by dietary frequency and preference data alone, it is hard to conclude whether children preferring and consuming these non-nutritious foods have greater susceptibility to acquire ADHD or that children with ADHD are commonly impulsive, without patience, and more likely seeking instant satisfaction and therefore favor hedonic low-nutrient-density foods [12].

The path analysis suggests that the nutrient-poor diet leads to suboptimal blood nutritional biochemistry which in turn contributes to the ADHD development. We found the B6, folate and B12 (B-vitamin factor) inadequacy were related to the lower intake of vegetable/fruit and protein abundant foods, and correlated to more intake of nutrient-poor foods. In addition, increased intakes of vegetable-fruit, flesh foods, and eggs contributed to a better iron status and lower phosphate concentration in blood, whereas nutrient-poor foods had an opposite effect. Being short of aforementioned whole foods and excessive in low-nutrient-density foods may compromise children’s vitamin and mineral status, and further link to ADHD characteristics. This also supports the potential inter-relationship among diet, biochemical profile, and behaviours among children. In the future, a clinical trial is warranted to untangle the direction of relationship; that is, to clarify whether modifying dietary pattern from nutrient-poor to nutrient-dense foods can correct children’s vitamin and mineral status, and further improve children’s ADHD symptoms.

Our study has some inherent limitations due to its nature of the case-control study design and bottle-neck in dietary data assessment. First, although we performed a SEM analysis, our study could not conclude the causal relationship between poor dietary patterns, nutritional status, and ADHD. Second, some may argue the inferior nutritional biochemistry profiles might result from the higher activity levels in ADHD children. However, we found the biochemistry profile is consistent with that food consumption assessed using the food frequency questionnaires. Compared to the control group, ADHD children preferred less nutrient-dense foods (i.e., vegetable, fruit, milk, meat, fish and eggs) and more nutrient-poor foods (Table 2). It is not clear whether it is due to the poor compliance of the ADHD children toward parental instruction to eat nutritious foods and hedonic response to the NP foods. However, since we also observe less preference toward nutritious foods in ADHD children (Table 2), we may infer that it is the food preference affecting their daily food choices and intakes which in turn may cause inferior nutritional status. Finally, we used the FFQ to assess the dietary profile of children, and the FFQ aimed to assess ones’ dietary intake in the previous year [23]. Our data showed that soymilk/egg intake was negatively correlated with n-6/n-3 fatty acid ratio. The main dietary sources of n-6 fatty acids were vegetable oils and the major cooking oil used in Taiwan, the soybean oil also contains gamma-linolenic acid, an n-3 fatty acid. It is hard to obtain accurate information on various fats and oils consumed by questionnaire in Asian countries, since cooking oils are added to dishes by the cook. Participants usually do not know the kinds of oils used and the amount added. In addition, the public cannot distinguish well between deep-sea fishes and those from non-salty water. The above-mentioned may explain why we could not find reasonable dietary contributors to the two fatty acid biochemical factors. Future research should further investigate abnormal fatty acid metabolism in ADHD and the relevant food choices.

## 5. Conclusions

The results of this study revealed that nutritional risk factors of ADHD include lower serum levels of B-vitamins as well as imbalance between iron status and inorganic phosphorus, between MUFA and SAF and between n-3 and n-6 PUFAs. Moreover, poor nutrition states may play a mediating factor between the link of unhealthy diet habit and ADHD. Further investigation is needed to clarify whether nutrition deficiency may contribute to abnormal brain function, and further lead to ADHD symptoms among children. Managing dietary and nutrition conditions may be trialed and considered as adjunct therapy for improving ADHD symptoms in children.

## Figures and Tables

**Table 1:** Demographic and anthropometric data, nutritional biochemistry, and frequency of food intakes between children with ADHD and healthy controls.

	Control (*N* = 216)	ADHD (*N* = 216)	
Demographic and Anthropometric Variables	Mean (SD)	Mean (SD)	*p*-Value ^a,b^
Age (years)	9.2 (1.8)	9.2 (1.7)	0.95
Male gender, n (%)	186 (86)	186 (86)	1
Height (cm)	135.2 (10.7)	135.2 (10.7)	0.93
Weight (kg)	33.6 (9.8)	33.6 (9.8)	0.95
Body mass index (kg/m^2^)	18.0 (3.1)	18.1 (3.1)	0.83
Father’s education	94 (43.5)	123 (56.9)	0.005 *
(High school or lower), n (%)			
Mother’s education	121 (56.0)	147 (68.1)	0.010 *
(High school or lower), n (%)			
Expenditure balanced with revenue (yes), n (%)	188 (87)	162 (75)	0.001 *
**Nutritional biochemistry**			
*Vitamins*			
Vit.B12 (pmol/L)	462.6 (151)	423.5 (150)	0.007 *
Folate (nmol/L)	19.6 (13.6)	15.3 (6.3)	<0.001 *
Vit.B6 (nmol/L)	58.7 (29.3)	49.9 (29.9)	0.002 *
*Minerals*			
Ferritin (ug/L)	44.7 (18.8)	39.9 (17.2)	0.006 *
Pi (mmol/L)	1.5 (0.2)	1.6 (0.2)	<0.001 *
Na (mmol/L)	147.1 (5.1)	146.5 (4.9)	0.21
K (mmol/L)	4.3 (0.3)	4.5 (0.3)	0.3
Ca (mmol/L)	2.4 (0.1)	2.4 (0.2)	0.15
Mg (mmol/L)	0.9 (0.1)	0.9 (0.1)	0.09
*Fatty acids*			
SFA (%)	40.7 (2.3)	41.2 (2.5)	0.023 *
MUFA (%)	26.4 (3.2)	25.4 (3.0)	0.001 *
PUFA (%)	32.9 (2.0)	33.4 (2.1)	0.022 *
n-6 FA/ n-3 FA ratio	8.7 (1.9)	9.4 (1.7)	<0.001 *
**Frequency of food intakes** (times per week)			
*Nutrient-dense foods*			
Vegetable	5.8 (1.6)	4.8 (2.6)	<0.001 *
Fruit	5.0 (1.8)	3.5 (2.6)	<0.001 *
Milk	4.2 (2.3)	3.3 (2.7)	<0.001 *
Yogurt	0.4 (1.1)	0.4 (1.0)	0.49
Meat	6.2 (1.0)	4.9 (2.1)	<0.001 *
Poultry	3.2 (2.0)	2.6 (2.2)	0.003 *
Pork	3.3 (2.2)	3.2 (2.1)	0.44
Beef	0.8 (1.4)	0.6 (1.1)	0.07
Fish	3.3 (2.3)	2.4 (2.1)	<0.001 *
Soy milk	1.0 (1.6)	0.6 (1.3)	0.01 *
Other soy products	1.8 (1.8)	1.4 (1.8)	0.06
Eggs	4.0 (2.0)	3.2 (2.3)	<0.001 *
*Nutrient-poor foods*			
Fried foods	0.9 (1.3)	1.4 (1.8)	0.001 *
Ice cream	0.6 (1.1)	0.9 (1.4)	0.004 *
Sugary, high-fat foods	2.2 (2.1)	2.6 (2.3)	0.07
High-fat snacks	0.9 (1.4)	1.3 (1.8)	0.02 *
Instant noodle	0.5 (1.1)	0.8 (1.4)	0.01 *
Sweetened beverage	1.9 (1.9)	2.9 (2.6)	0.001 *
Shaved ice desserts	0.5 (1.0)	0.8 (1.5)	0.005 *
Candy and chocolate	1.3 (1.5)	1.7 (2.0)	0.02 *

* *p*-value < 0.05; Chi-square test for groups comparisons of binary variables and *t*-test for groups comparisons of continuous variables.

**Table 2:** Principal components identified from blood nutritional biochemistry markers and from food frequency ^a,b^ and factor loading scores of the studied variables.

Blood Nutritional Biochemistry Markers	Factor 1: MUFA/SFA Fatty Acids	Factor 2: B-Vitamins	Factor 3: Minerals	Factor 4: n-6/n-3 FA Ratio
Vit.B12 (pmol/L)	−0.066	**0.546**	0.156	−0.058
Folate (nmol/L)	0.085	**0.807**	0.061	−0.043
Vit.B6 (nmol/L)	0.120	**0.731**	−0.069	0.107
Ferritin (ug/L)	0.084	0.103	**0.768**	0.175
Pi (mmol/L)	0.129	−0.037	**−0.734**	0.161
SFA (%)	**−0.932**	−0.024	−0.003	0.130
MUFA (%)	**0.929**	0.092	−0.059	0.072
n-6/n-3 FA ratio	−0.054	−0.010	0.008	0.968
**Food frequency** (times per week)	**Factor 1: Nutrient-Poor Foods**	**Factor 2: Vegetable-Fruit**	**Factor 3: Flesh foods**	**Factor 4: Soymilk-Egg**
Vegetable	−0.126	**0.795**	0.176	−0.043
Fruit	−0.074	**0.819**	0.154	0.094
Milk	−0.112	0.204	0.275	−0.041
Meat	−0.036	0.211	**0.795**	0.042
Poultry	0.191	−0.163	**0.704**	0.025
Fish	0.043	0.258	**0.560**	0.263
Soy milk	0.052	−0.097	−0.066	**0.827**
Eggs	−0.051	0.130	0.258	**0.610**
Fried foods	**0.583**	−0.285	0.191	−0.172
Ice cream	**0.748**	0.018	−0.009	−0.038
High-fat snacks	**0.646**	−0.195	0.085	−0.004
Instant noodle	**0.604**	−0.247	0.072	0.036
Sweetened beverage	**0.505**	−0.321	0.024	−0.024
Shaved ice desserts	**0.744**	0.161	−0.053	0.086
Candy and chocolate	**0.616**	0.045	−0.124	0.049

^a^ Rotation method was Varimax with Kaiser Normalization. ^b^ Variables (boldface) with factor loading score more than 0.50 are regarded as main contributors to components and served as further analysis.

**Table 3:** Correlation between the four nutritional biochemistry factors and the four dietary factors.

	MUFA/SFA		B-Vitamins		Minerals		n-6/n-3 FA Ratio	
	*r*	*p*-Value	*r*	*p*-Value	*r*	*p*-Value	*r*	*p*-Value
**Nutrient-Poor Foods**	0.043	0.373	−0.235	<0.001 *	−0.205	<0.001 *	0.009	0.845
**Vegetable-Fruit**	0.039	0.414	0.217	<0.001 *	0.195	<0.001 *	−0.057	0.236
**Flesh Foods**	0.007	0.883	0.099	0.039 *	0.110	0.022 *	0.012	0.801
**Soymilk-Egg**	0.028	0.561	−0.004	0.938	0.116	0.016 *	−0.119	0.013 *

* *p*-value < 0.05; *r* = Pearson Correlation.

## Supplementary Materials

The following are available online at https://www.mdpi.com/2077-0383/8/5/709/s1, Table S1: Food preference between children with ADHD and healthy control children; Table S2: The relationship between ADHD, the dietary factors and the nutritional biochemistry factors.
