# Supplementary material for: Dietary Profiles, Nutritional Biochemistry Status, and Attention-Deficit/Hyperactivity Disorder: Path Analysis for a Case-Control Study
Source: J Clin Med. 2019 May 18;8(5):709. doi: 10.3390/jcm8050709 (PMC6572510; doi:10.3390/jcm8050709)
Supplement: Supplementary file 1 [file jcm-08-00709-s001.pdf]

Supplementary Table 1

Food preference between children with ADHD and healthy control children

	**Control (N=216)**	**ADHD (N=216)**	**t**	**P-value**
Vegetables	2.64 ± 0.58	2.25 ± 0.82	-5.722	<0.001
Fruit	2.85 ± 0.38	2.79 ± 0.51	-1.494	0.136
Milk	2.75 ± 0.56	2.48 ± 0.81	-4.078	<0.001
Yogurt	2.58 ± 0.67	2.25 ± 0.86	-4.498	<0.001
Yakult	2.70 ± 0.59	2.67 ± 0.64	-0.548	0.584
Cheese	2.13 ± 0.81	1.94 ± 0.92	-2.278	0.023
Meat	2.65 ± 0.54	2.37 ± 0.75	-4.417	<0.001
Poultry	2.42 ± 0.77	2.25 ± 0.86	-2.173	0.030
Pork	2.25 ± 0.83	2.13 ± 0.91	-1.546	0.123
Beef	2.44 ± 0.76	2.25 ± 0.91	-2.347	0.019
Fish	2.65 ± 0.65	2.53 ± 0.76	-1.838	0.067
Seafood	2.33 ± 0.84	2.51 ± 0.79	2.250	0.025
Visceral	1.71 ± 0.86	1.60 ± 0.86	-1.282	0.201
Soy Milk	2.52 ± 0.72	2.32 ± 0.87	-2.667	0.008
Bean Products	2.46 ± 0.74	2.41 ± 0.81	-0.683	0.495
Eggs	2.76 ± 0.51	2.75 ± 0.58	-0.353	0.724
Hamburg	2.36 ± 0.80	2.68 ± 0.51	4.907	<0.001
Ice Cream	2.61 ± 0.65	2.77 ± 0.46	2.981	0.003
Sweet High Oil Food	2.55 ± 0.66	2.68 ± 0.58	2.176	0.030
Sweet High Oil Food	2.40 ± 0.75	2.65 ± 0.55	3.885	<0.001
Instant Noodles	2.34 ± 0.79	2.56 ± 0.64	3.094	0.002
Biscuits	2.51 ± 0.68	2.55 ± 0.69	0.492	0.623
Soda	2.53 ± 0.70	2.77 ± 0.48	4.204	<0.001
Popsicle	2.53 ± 0.70	2.77 ± 0.48	4.204	<0.001
Candy	2.35 ± 0.79	2.68 ± 0.55	4.931	<0.001

Supplementary Table 2

The relationship between ADHD, the dietary factors and the nutritional biochemistry factors

	ADHD	Father's education	Mother's education	Family Expenditure
**Dietary factors**				
Nutrient-poor foods	0.002	0.427	0.152	0.001
Vegetable-fruit	<0.001	0.008	0.408	0.030
Protein	<0.001	0.176	0.358	0.764
Egg	0.001	0.444	0.773	0.861
**Nutritional biochemistry factors**				
Fatty acids	0.012	0.313	0.313	0.612
Vitamins	<0.001	0.256	0.499	0.065
Minerals	<0.001	0.002	0.376	0.951
n-6/ n-3 FA ratio	0.001	0.240	0.233	0.722

Statistical values were estimated using the General Linear Model, controlling for parents' education levels and family expenditure.
